# High throughput application of ASTM D8332: Detailed prototype design and operating conditions for microplastic sampling of riverine systems

**DOI:** 10.1016/j.mex.2024.102680

**Published:** 2024-03-26

**Authors:** Jeremiah Bryksa, Patric McGlashan, Nadia Stelck, Jon Wong, Andrew Anderson-Serson, Matthew Hart, Trace Malcom, Bob Battle, Paolo Mussone

**Affiliations:** Northern Alberta Institute of Technology, 10210 Princess Elizabeth Ave, Edmonton Alberta, T5G 0Y2 Canada

**Keywords:** Microplastics, Freshwater, Environmental sampling, Plastic pollution, NAIT High Throughput Microplastic Sampling

## Abstract

Microplastic sampling strategies for aquatic systems commonly employ small mesh nets to collect suspended microparticles. These methods work well for marine sampling campaigns; however, complex water systems such as freshwater rivers, effluent discharges, and stormwater ponds characterized by high total suspended solids and fast-moving water can cause the nets to clog, rip, or tear. Published in 2020, ASTM D8332 is an alternative approach to sampling complex water systems for microplastics involving pumping large volumes of water across a cascading stack of sieves to collect suspended particles. Here we show that ASTM D8332 can be applied to sample freshwater rivers for microplastic collection. A high throughput sampling prototype developed in this work is capable of pumping 1500 L of river water in 45 min to collect particles as small as 45 µm. The system is lightweight, modular, and easily transportable. It has a discrete power supply, allowing for the collection of microplastics anywhere along the river, including municipal discharges. The design minimizes the amount of plastic in the flow path and provides a practical way to measure field contamination. Finally, we outline lessons learned through extensive field trials and testing using this system sampling the North Saskatchewan River in Edmonton, Alberta.

• Existing small mesh nets face limitations in freshwater rivers, encountering clogging and tearing issues from high suspended solids and fast moving water.
• Using a standardized method, ASTM D8332 - a pumping-based approach is efficient for microplastic collection in freshwater rivers.
• Lightweight, modular, plastic free prototype system pumps 1500 L of river water in 45 min, collecting particles as small as 45 µm. Successfully tested in the North Saskatchewan River.

## Graphical abstract

**Figure:**
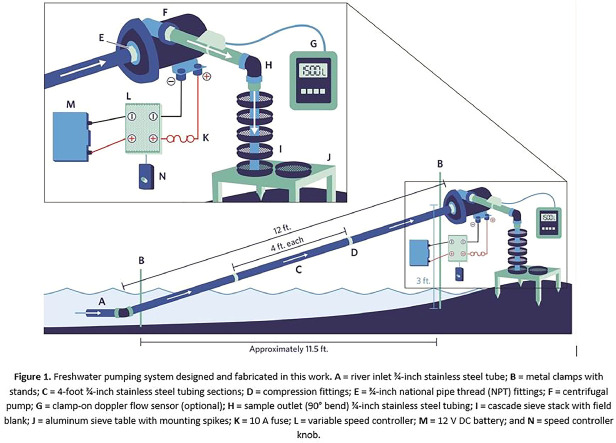

Specifications table

**Table:** 

Subject area:	Environmental Science
More specific subject area:	Microplastic Fresh Water Sampling
Name of your method:	NAIT High Throughput Microplastic Sampling
Name and reference of original method:	ASTM: D8332-20 Standard Practice for Collection of Water Samples with High, Medium, or Low Suspended Solids for Identification and Quantification of Microplastic Particles and Fibers
Resource availability:	Materials: • Centrifugal pump: Jabsco 12 V • Lithium-iron phosphate battery: Expert-Power 20 Ah, 256 Wh • ¾′′ OD stainless steel tubing • Assorted NPT fittings and 90° elbows • Variable speed controller and associated electrical components • Waterproof enclosers • ASTM E11 / ISO 31,310 8′′ stainless steel sieves (5 mm, 500 µm, 125 µm, and 45 µm) • Dual slot aluminum sieve table • Hiking equipment, chest waders, personal flotation device • Securing stands and clamps

## Method details

### Background

Microscopic plastic particles are present throughout the environment. This plastic microlitter, termed microplastics, have been found in every ocean [1,2], in rivers [3], [4], [5], and in organisms throughout the ecosystem [1,[6], [7], [8], [9], including humans [10]. The consequences of microplastics in the environment and long-term health effects on organisms are an active area of research and a growing concern for many governments [11]. Microplastic collection, identification, and quantification are technically challenging [12], [13], [14]. This paper focuses on aquatic sampling and aims to provide a detailed prototype design and operating conditions for sampling microplastics in complex riverine freshwater systems.

Standard sampling methods for water matrices employ mesh nets to filter out microplastic particles. Methods have been adapted and optimized to sample oceans using plankton, neuston, and mantra nets [14], [15], [16], [17]. Mesh nets are very successful in collecting microplastic samples from marine environments as they allow researchers to sample large surface water areas with relative ease of deployment and operation [17,18]. Mesh nets have been used to collect the majority of marine microplastic samples, often in the size of 300 µm [15,^16^,^19^,20]. Oceans have been the most studied water matrix [21]; however, there is a direct need to study more complex water systems such as rivers, wastewater treatment plant effluent discharges, and stormwater ponds [22], [23], [24] because these constitute source points and vectors for microplastics transport to oceans. There are challenges with employing mesh nets in these water systems because there is an increased risk of the mesh ripping and clogging with water that has high suspended solids loads or a high velocity/flow rate [25]. Importantly, there is a lower size limit that can be practically sampled with mesh nets, even in marine environments [14,26]. Because of this, there is a significant knowledge gap in microplastic occurrence below 100 µm in size [14,27].

One alternative water sampling method is cascade sieving, which involves filtering bulk quantities of water through a stack of sieves with discrete mesh sizes. The cascading effect of the water across the sieves decreases the likelihood that any single sieve may become clogged. This technique offers advantages over a collection with mesh nets in that it is capable of in-situ size fractioning, increased accuracy regarding the total water volume sampled, and can sample a lower size range of microplastics that mesh nets cannot practically sample [13,[28], [29], [30]. Also, systems have been designed that effectively control contamination [31]. Sieve filtration methods have progressed over the years, beginning with researchers pouring 10 L bucket grab samples in multiple increments over the sieves [32] to pumping water using electric [33,34], peristaltic [31], submersible [29], and diaphragm pumps [35,36]. Efforts to correlate mesh net sampling with pumping methods have been attempted but more research is needed [29,30]. Successful pump sampling methods have been employed in the wastewater industry to attempt to understand microplastic concentrations [37,38]. Researchers have shown that large-volume water samples can be taken using cascade sieving, collecting microplastics as small as 20 µm [21,^28^,^33^,39].

Another challenge microplastic researchers face is the lack of standardized methods for the collection and analysis of microplastics. The publication of ASTM D8332 [40] is a direct solution to this problem, enabling microplastic analytical work to be executed uniformly, generating data that can be compared between studies [12,14]. Our contribution demonstrates that ASTM D8332 can be applied to river settings, therefore expanding its range of applicability. For this study, we selected the North Saskatchewan River, one of western Canada's most important rivers, and used the method to carry out a study about microplastics occurrence within the boundaries of the Edmonton Municipal Region, the largest urban and industrial cluster in its watershed. The North Saskatchewan River can have high total suspended solids and varying flows due to seasonal run off and varying rain events throughout the year. We provide a detailed overview of our sampling prototype design, component summary, and the rationale for their selection. We have optimized field operating conditions over two field seasons and offer lessons learned in this contribution. Some literature is available demonstrating that cascade sieving has been attempted in river sampling campaigns, but this body of knowledge is limited to data generated using low volumes of water filtered [34], [35], [36]. Our high throughput microplastic sampling prototype allows for the filtering of large volumes of river water (1500 L) in 45 min to collect microplastics as small as 45 µm. The system can fit into hiking backpacks and has its own discrete power supply, thus allowing it to be set up anywhere where sampling is desired. Finally, the system has a plastic free-flow path and is designed to operate semi-autonomously. This minimizes field contamination, and we provide a practical solution to collect representative field blanks to ultimately quantify any field contamination present.

### Design considerations for prototype freshwater sampling system

A novel freshwater sampling system for microplastics evaluation was designed with the following key features in mind: 1) Lightweight and portability, two factors crucial for success of any microplastic project designed around allowing remote sampling, especially when sampling points along the river valley are only accessible by foot. Some of the sampling sites in this work were a 20-minute walk from vehicle parking with marginal trails, so lightweight, modular components were chosen to overcome this challenge. The system was composed of modules that fit into backpacks for easier transportation. 2) Modularity, allowing for ease of assembly and disassembly on site, to thus enable access to multiple sampling sites per day. One key advantage of our design was that it enabled the team to safely traverse rough terrain, free of carrying heavy or bulky equipment, providing access to the shoreline regardless of steep egresses. Importantly for most sites, especially the remote sites outside of the city limits, there was no existing, reliable sampling infrastructure in place. 3) Discrete and reliable power source so that sampling could be carried out in situ. 4) Exclusively plastic-free components to overcome contamination issues, one of the most challenging aspects of microplastic science. This was challenging to achieve because most commercially available equipment or components have some form of plastic in them. We took particular care to ensure that we minimized the presence of plastic components in all aspects of our pumping system's design, especially the flow meter and pump, while stainless steel tubing was chosen as the main plumbing material. Having a completely rigid tube plumbing system complicated the experimental setup, specifically for the initial pump priming step, but to our advantage, stainless steel offers strength and field ruggedness to the system.

**Fig. 1:**
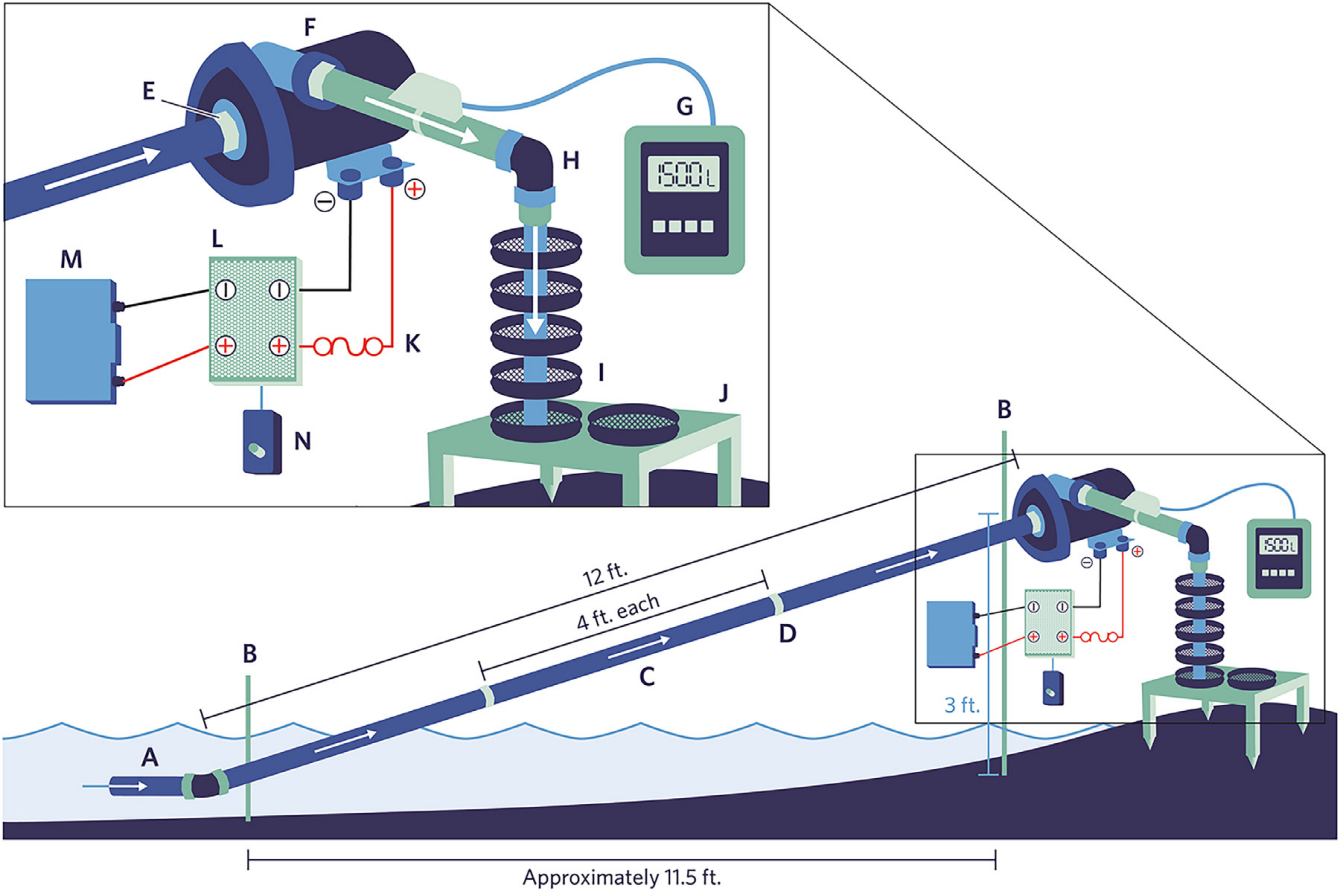
Freshwater pumping system designed and fabricated in this work. **A** = river inlet ¾-inch stainless steel tube; **B** = metal clamps with stands; **C** = 4-foot ¾-inch stainless steel tubing sections; **D** = compression fittings; **E** = ¾-inch national pipe thread (NPT) fittings; **F** = centrifugal pump; **G** = clamp-on doppler flow sensor (optional); **H** = sample outlet (90° bend) ¾-inch stainless steel tubing; **I** = cascade sieve stack with field blank; **J** = aluminum sieve table with mounting spikes; **K** = 10 A fuse; **L** = variable speed controller; **M** = 12 V DC battery; and **N** = speed controller knob.

We successfully minimized the number of plastic components used while achieving the goal of designing and fabricating a lightweight, portable system capable of sampling fresh water for microplastics that is based on the principles of ASTM D8332. The as-designed and produced prototype microplastics sampling system is illustrated in Fig. 1.

### Method application, adaptation, and optimization

ASTM D8332 was first published in July 2020 and has not included any revisional updates since then. Some adaptations to the specifications of ASTM D8332 were necessary for this prototype system. Based on our experiences in field settings, some variations to the specifications of ASTM D8332 are suggested for consideration. Additionally, during our initial field trials, we encountered unforeseen challenges that we had not anticipated in the design phase of the project. We outline here some of those challenges and propose solutions to resolve them. The purpose of presenting our trial-and-error approach is to serve as a learning opportunity for any other research group either considering designing an alternative microplastics sampling system based on ASTM D8332 principles or adopting our sampling method to suit other specific microplastics sampling needs in the field.

**Pump.** The pump was the critical element of the sampling system because it needed to be capable of pumping enough fresh water across a cascading sieve stack to collect suspended particles in the river and provide a representative sample volume for microplastics. While Section 6.6.1 of ASTM D8332 outlines the use of a submersible pump, we decided to use a centrifugal pump for the reasons outlined below.

One major disadvantage of most submersible pumps is that they operate on 120 V AC. This was a limitation when designing the power delivery system and the electronics for our microplastic sampling system. Because our system is battery powered, we needed to consider the pump and available battery options together to meet the portability criterion, and no readily available 120 V AC battery option enabled this. A submersible pump may be a good choice if there is an easily accessible plug-in power source, e.g., from a nearby building, or if a generator could be used, but this would detract from achieving portability requirements. Other pump options considered included diaphragm pumps and peristaltic pumps, but we were unable to find a suitable diaphragm or peristaltic pump that met our compatibility requirement because all pumps had multiple forms of internal plastic components, plastic check valves, and rubber tubing and seals and most were very slow. Of the options considered, we deemed the Jabsco 12 V AC freshwater pump as the best suited for our prototype microplastics sampling system. It can pump large quantities of water, it contains a minimal number of plastic components (other than a rubber seal in the main body of the pump), it can be powered by a 12 V DC battery, and it is well suited to handle water containing solid particles.

Another disadvantage of having a centrifugal pump is that it needs to be primed because it cannot operate from a dry state. The pump needs enough water backpressure to initiate the centrifugal pumping process and the system needs to be free of air. This is more of a procedural adaptation and priming can easily be achieved by following our protocol.

Our prototype and sampling method deviates from ASTM D8332 in one additional way. As seen in *ASTM D8332* Fig. 1 *Water Sampling Apparatus for Non-Pressurized Systems*, the submersible pump is on the inlet side of the system, whereas in our system we have the centrifugal pump on the outlet side. In our sampling protocol we used stainless steel stakes and clamps to fix the river inlet and the centrifugal pump in one orientation. The inlet faces the river current, and the pump is on the shore side, hovering over the sieve stack. Having a fixed in-place, rigid system is advantageous because it produces a very constant and uniform flow rate; however, one disadvantage of the setup is that it can be susceptible to stalling when the centrifugal pump sucks in air. This limits the position of the river inlet to approximately 1–1.5 inches below the surface. Having the river inlet any closer to the surface leads to an increase in the likelihood it will suck in air from the river surface's natural turbulence. If this occurs, the sampling process must revert to the system priming step. This would add time to the sample collection process, create an interrupted sample, and add to the time field staff spend proximal to the pump and sieve stack, leading to an increased risk of contamination from fibers.

**Battery.** We initially trialed the use of lithium deep cycle batteries because they have a stable charge capacity, allowing for many charge–discharge cycles. Lithium-iron-phosphate batteries were included in the final design because they were the lightest weight option available. This 12-volt option can power the centrifugal pump for multiple 1500 L samples. It was housed in a waterproof protective case to offer extra protection while working near the river's edge.

**Plumbing.** The entire system was plumbed with ¾-inch (OD) stainless steel tubing assembled using 4-foot sections. ¾-inch tubing was chosen to eliminate possible limitations of our flow rates, and because the Jabsco freshwater pump has ¾-inch NPT connections. The 4-foot sections of stainless-steel tubing could be assembled on-site, via compression fittings, depending on how far from shore the samples need to be taken from. This modularity of the system's plumbing aligned well with our portability criterion. Typically, 3 sections (12 feet) were used to sample the surface water. The stainless-steel tubing could be easily rinsed after use because it is just a straight pipe. The sample inlet consisted of a 90° elbow from the main sampling path that always faced into the river flow. A 5-mm mesh screen could be added to the inlet if there is much debris in the river at the time of sampling. On the outlet side, another 90° bend allowed the water to flow onto the sieve stack, collecting the suspended particles.

Before settling on the use of rigid tubing we field-trialed the use of corrugated stainless-steel tubing. The corrugated tubing provided a degree of flexibility while meeting our material compatibility criterion. This was advantageous for the set-up phase on-site, making it easier to position the inlet and outlet. However, we quickly realized that the internal structure retained a small number of particles, regardless of cleaning and flushing efforts. Because this could lead to cross-contamination from site to site, we opted to scrap this from the final design.

**Flow Meter.** We had difficulty obtaining an inline flow meter that met our material compatibility requirement. Additionally, we desired that the meter run off its own power source and not be wired into the main battery for simplified operations. Sampling conditions should also be considered. A select number of candidates were available, but a commonality for these inline flow meters is that they operate using impellors. The suspended solid load within the North Saskatchewan River includes small blades of grass that can easily be sampled with the centrifugal pump. These could potentially wrap around the flow meter impeller, causing it to fall out of calibration and rendering it unusable.

Manual flow rate determination is the easiest method for measuring flow rate in the field. This can be done by using a known volume bucket with a timer to determine flow rate. Once the system is set up and fixed in place with metal clamps and stands, the flow rate does not change substantially as the height between the river inlet and sample outlet remains constant. Our method for accurately sampling fixed volumes of river water for microplastics is to determine flow rate at the start of the sampling process and calculate how long it takes to pump the total desired volume, which is usually 1500 L. This fixed volume bucket calibration method adheres to the accuracy statement of ASTM D8332, which is +/−5 % total volume sampled.

**Fig. 2:**
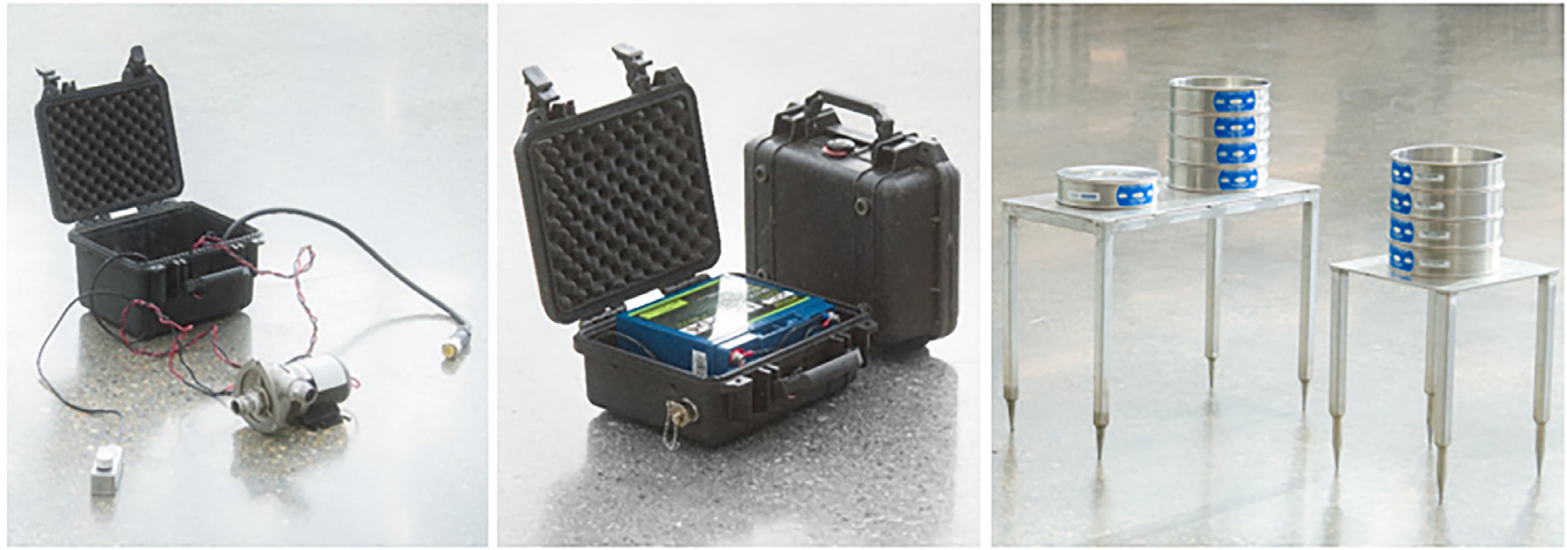
High throughput microplastic sampling system components: centrifugal pump with electronics housed in a protective case (left), lithium iron phosphate battery housed in a protective case (center), cascading sieve stacks for samples and blanks with aluminum tables (right).

**Fig. 3:**
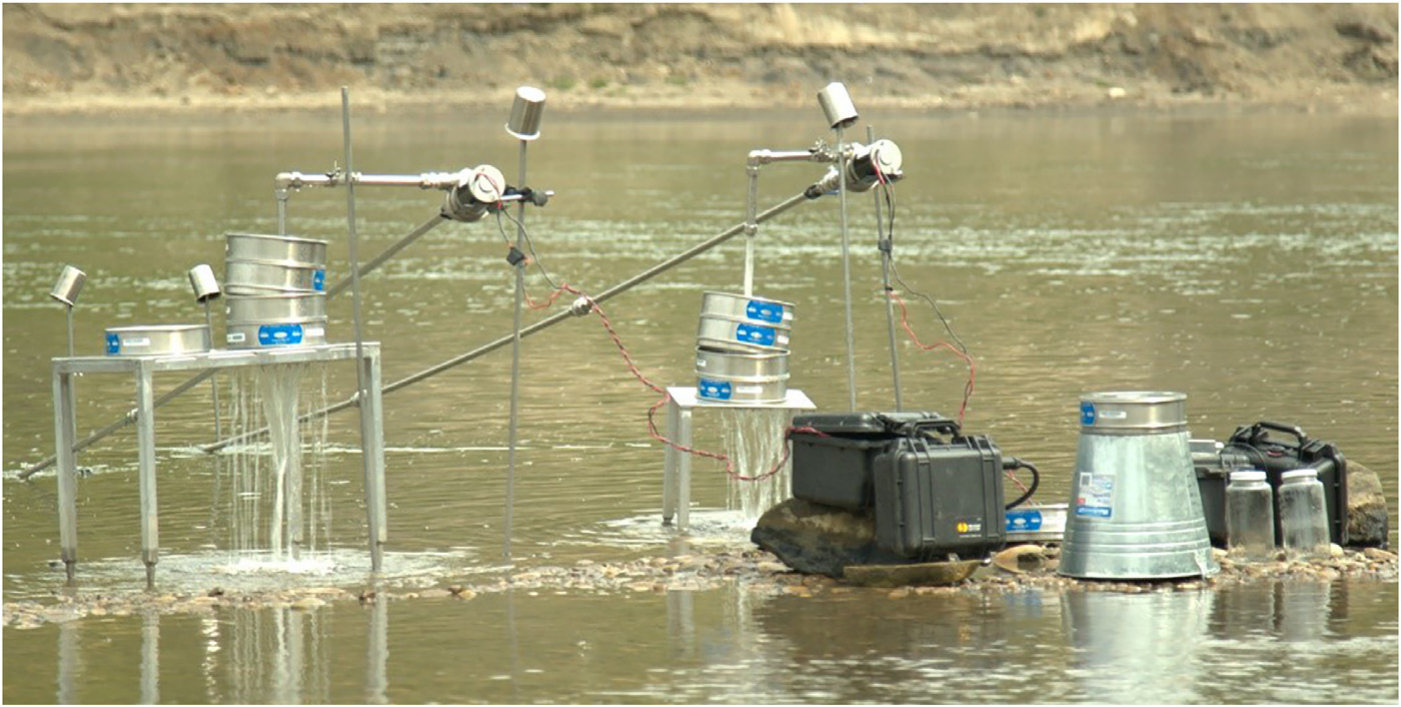
Pumping river water in the field through a cascade sieve stack using 2 high throughput microplastic sampling system.

**Fig. 4:**
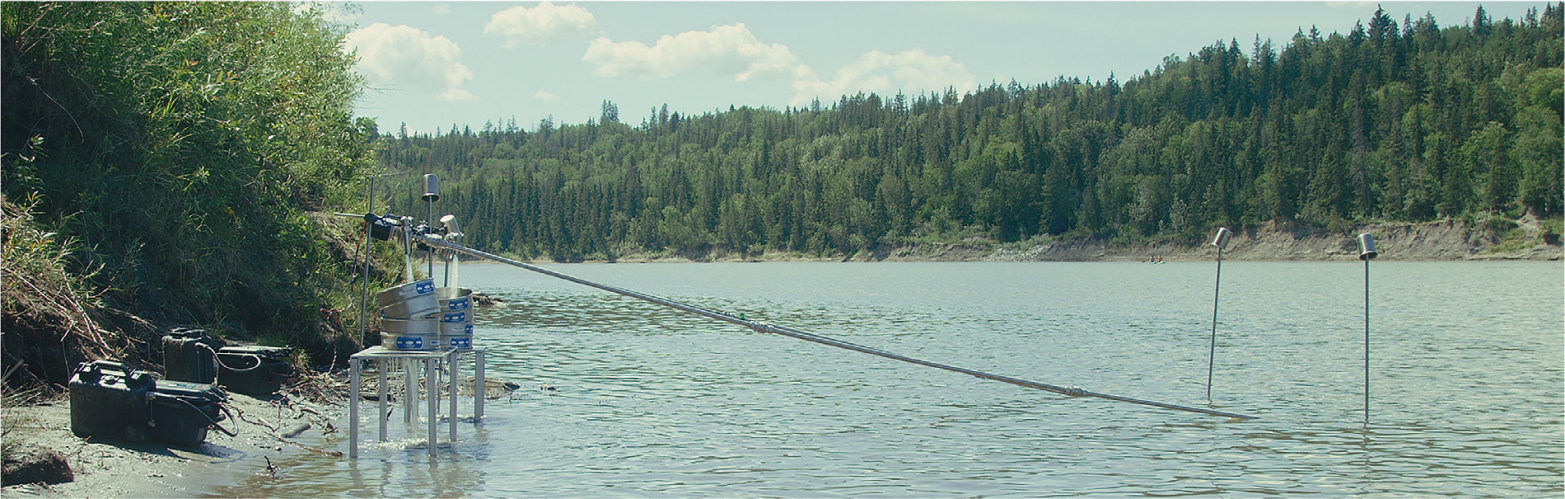
Duplicate set-up of NAIT high throughput microplastic sampling system on the North Saskatchewan River.

Another available option is using the Greyline PDFM 5.1 portable doppler flow meter that can be clamped external to the sample flow path (Fig. 1.). This allows for the flow rate to be accurately measured using particle transient time calculations and doppler sonar without the need for an inline measurement. Additionally, the totalized flow can be displayed over time. The system can be mounted on the outside of the outlet with ¾-inch stainless-steel plumbing to send sonar into the tube, allowing for an accurate real-time calculation of flow (after being corrected for the inner diameter and determining the optimal distance from the bends in the tubing). The portable doppler flow meter is a suitable option for flow monitoring; however, it is an expensive option, and it takes considerable time to set up in the field to read accurately. This one component of the sampling system can cost more than all the other components combined. Fig. 2, Fig. 3, Fig. 4

**Sieve Stack.** The sieves used to collect the suspended particles were purchased through a local laboratory supply vendor and conformed to ASTM E-11 specifications. At the start of the 2021 field season, we used the following size sieves: 5 mm, 1 mm, 500 µm, 300 µm, 125 µm, and 45 µm. Throughout that field season, we modified the sieves used because some were almost empty after 1500 L of sampling, particularly the 1 mm and 300 µm sieves, which we then removed from our process workflow. The 45 µm sieve was chosen as our lower size limit to match our laboratory and instrumentation process, which was optimized and validated for microplastics as low as 45 µm. Sieves were stacked on top of each other as per Section 6.5.2 of ASTM D8332. The sieves sat on an aluminum table that was fabricated to exactly fit the 8-inch standard size sieves and that had spiked legs to help stabilize it along the riverbed.

The cascading effect of particles filtering down the sieve stack prevented the smallest sieve from becoming clogged immediately, although clogging did occur as the North Saskatchewan River has a considerable total suspended solids content. Based on this, we recommend having a spare 45 µm sieve that can be swapped out to avoid halting the sample collection and losing the prime while maintaining sample consistency. The clogged 45 µm sieve could be rinsed into sample containers and reused if clogged again. After a sample was collected, using stainless steel beakers, sieves were extensively rinsed in the field using filtered water from the bottom of the sieve stack into glass containers with aluminum lids. After each field day the sieves were cleaned by cycles of sonication and rinsing with filtered type 2 water. On site, before each sampling event, the sieves were pre-rinsed using site water to clean any contaminants from the transportation process. Visual inspection should also be conducted regularly to check for damaged mesh, especially the 45 µm sieves. We replaced all 45 µm sieves every 2 months as a precautionary measure.

**Field Sampling Blanks.** Field blanks are not addressed in ASTM D8332. However, from the literature and based on our experiences, we believe it is crucial to quantify this value as it is impossible to avoid microplastic contamination in the field. Sources may include atmospheric contamination, people in the sampling vicinity, and contamination from field clothes and personal protective equipment (PPE). The sampling team ensured no synthetic fibers were worn to the field, but our safety protocol requires staff to wear personal flotation devices (PFDs) near the river and hats to protect from the sun. These PPE items were made from nylon and polyester, both of which were analytes for our investigation.

To obtain an accurate and representative field blank we designed the sieve table to have an additional slot to hold a blank sieve. A separate 45 µm sieve was precleaned according, as above, and wrapped with tin foil to avoid contamination until sampling began. Once sample collection commenced, the blank sieve was unwrapped and placed in the second slot in the sieve table and left for the duration of the sample collection. Care was taken by the field team to remain down wind of the sieve stack and minimize the amount of time spent near the sampling device. The system was operated with minimal operator's input after set up, other than monitoring for any clogging.

### Testing under field conditions

The prototype microplastics freshwater sampling system was designed by the NAIT Technology Access Centre for Sensors and System Integration (TACSSI) in the winter months of 2020–2021, following the initial publication of ASTM D8332 in summer 2020. In the spring of 2021, the system was ready for deployment for further testing in the field.

The system required a minimum of two people to operate it because one person needed to be on the river inlet side for priming and one person needed to be on the shore operating the outlet end and power controls. Once the system was fully primed and pumping it could be operated autonomously. This was of significant advantage because the field staff could stand relatively far from the system, downwind, to limit the possibility of sample contamination.

The system could operate at approximately 33 L/min (ca. 9 Gal/min) allowing us to collect a 1500 L sample in approximately 45 min. We wanted to prove that an expensive flow meter is not required. We verified our fixed volume bucket calibration method in the field using a 24 V AC *Endress Hauser Picomag Electromagnetic* inline flow meter that was installed inline at an optimal distance from turbulent flow. This inline flow meter was chosen for verification because it had a certificate of calibration but would never be used under sampling conditions because of its internal plastic components and power requirements. For this experiment this meter was externally powered by a generator. Determining the flow rate initially and then applying a calculation to determine the pumping time to reach 1500 L we evaluated our totalized flow error to be 3.5 %. We performed this experiment 5 times (*n* = 5) for a total of 7500 L pumped. This difference in totalized flow is within the ASTM D8332 specified maximum allowed error of 5 %. We speculate that the low bias is caused by battery charge decay over the sampling period. We operate the pump at maximum flow, and as the battery loses charge the maximum flow is decreased. We have minimized this concern by using a fresh battery, with full charge, for every sample taken.

To test if the high throughput flow rates and large volumes of water cascading across the sieves were pushing particles through the 45 µm sieve (that should normally be retained) we performed a breakthrough experiment. Because the sampled river has high total suspended solids, we used these particles as a proxy for microplastics to determine if particles are passing through the smallest sieve by installing a second 45 µm sieve below our normal sieve stack and comparing the total particles on the duplicate 45 µm sieves. We performed this experiment 6 times (*n* = 6) and using Eq. (1) we determined that ≤1 % of particles broke through the first 45 µm sieve.

(1) $$Breakthrough\;\%=\left(\frac{\text{mg}\;\text{solids}\;\text{in}\;\text{bottom}\;45\;\mu m\;\text{sieve}\;}{\text{mg}\;\text{solids}\;\text{in}\;\text{top}\;45\;\mu m\;\text{sieve}+\text{bottom}\;45\;\mu m\;\text{sieve}}\right)\times \;100$$

We carried out the North Saskatchewan River microplastics sampling approximately 12 feet from the shore. This was achieved by adding multiple 4-foot sections of ¾-inch stainless steel tubing, connected via compression fittings. This distance was chosen to accommodate river depth. Our aim was to sample in depths of at least 2 feet of water because any shallower would increase the probability of the pump sucking up riverbed debris or rocks. We also wanted to ensure there was enough river flow, the first couple of feet from the shore had stagnant water, and we wanted to ensure we were getting a representative sample of the river. Tubing sections were connected tightly so that the pump could achieve a proper prime. If the pump was not maintaining flow the first troubleshooting step was to tighten the compression fittings.

The system was designed to be operated in all spring, summer, and fall weather conditions including rain. The two protective cases that housed the electronics for the pump and battery were IP 67 rated. This was of particular importance for the lithium-iron-phosphate battery. We designed the power connections with safety in mind and built the circuit to have a weather-resistant threaded power connection to add more protection from the elements. The pump was wired with long positive and negative cables allowing the pump to hover over the sieve stack when sampling, while the electronics were safely housed in a waterproof case. The pump was field tested and proven to be weather resistant and able to operate in rainy conditions. This was important for providing an ability to study urban runoff by enabling field teams to sample during a storm event.

### Limitations and future improvements

There are three notable limitations with this method. Regarding freshwater sampling, one requirement is that the body of water needs sufficient flow and depth to accurately pump 1500 L. This can be problematic in situations where the river has decreased flow or insufficient depth to allow for inlet submersion due to seasonal drought. This also compounds when considering the drainage water from the sieve stack. If there is insufficient flow to carry the drainage away from the inlet it can lead to sample contamination.

The second limitation is that the current prototype is unable to completely sample the surface water because air in the system will stall the centrifugal pump as outlined above. We are currently working on a new inlet design to solve this problem which we plan to test in the field during the summer of 2023. At this juncture, we recommend placing the inlet of this system at a sampling depth of at least 1 inch below the surface (or lower if necessary).

The final limitation of this method is the difficulty to completely rinse the 45 µm sieves into the sample bottles. The pores for these are very small and easily clogged. A small number of particles from the river can get trapped in these pores and remain on the sieve even after extensive rinsing with water. This means a small portion of the sample is left behind on the sieves. Proposed solutions could be using sieve brushes or rubbing the sieves to dislodge the stuck particles. We have opted to reject these due to the potential for contamination. Tools such as a sonicator are impractical to bring to the field but the sieves could be brought back to the lab for sample transfer if desired.

In the supplementary material we propose a new wiring scheme. The original design has a DC motor with a variable speed controller to adjust the speed of the pump. In practice this functionality was never used, in most cases we ran the pump at maximum speed. The motor speed controller has been replaced by a DC-DC converter to provide a constant 12 V to the pump regardless of the battery voltage. This new design should address the flow rate decay related to battery charge and help improve the accuracy of the sampling system.

One improvement that could be made to the system is finding a suitable inline flow meter that is plastic free and battery powered. As mentioned before, some impeller style meters are being considered. To minimize the likelihood that grass or other material can damage the impeller we may consider using them only at the start of the sampling process to determine initial flow rate, replacing the manual flow rate determination. Having a totalized flow that can be measured throughout the sampling process should improve the accuracy of this method.

### Conclusions

Freshwater river systems like the North Saskatchewan River pose unique challenges for microplastic sampling due to highly variable seasonal flow rates and high total suspended solids loads which increase the risk of ripping and clogging commonly used microplastic sampling mesh nets. ASTM D8332 offers a solution to this problem by suggesting a pumping system to filter water through a stack of cascading sieves to collect the microplastic particles.

We designed a freshwater pumping system that 1) is lightweight and portable, 2) is simple and modular, 3) is capable of operating on battery power, and 4) does not contain any plastic materials. The system was thoroughly tested, and modifications were made to optimize the system to collect microplastics from the river while maintaining portability and component compatibility. Some modifications to ASTM D8332 were necessary, including changes to the plumbing, modifying priming operations, and optimizing flow and sampling conditions.

This newly developed prototype system was used to collect over 100 freshwater samples along the North Saskatchewan River, spanning over 100 km of the river in the spring, summer, and fall months of 2021. The system is weather resistant and can operate in all spring, summer, and fall sampling conditions. The system is capable of pumping 1500 L of fresh water within 45 min and can collect microplastic particles from the river in the range of 5 mm–45 µm, while allowing for field sampling blanks to be collected.

## CRediT authorship contribution statement

**Jeremiah Bryksa:** Conceptualization, Methodology, Validation, Investigation, Writing – original draft. **Patric McGlashan:** Validation, Investigation. **Nadia Stelck:** Validation, Investigation. **Jon Wong:** Validation, Investigation. **Andrew Anderson-Serson:** Conceptualization, Methodology. **Matthew Hart:** Conceptualization, Methodology. **Trace Malcom:** Conceptualization, Methodology. **Bob Battle:** Supervision. **Paolo Mussone:** Writing – review & editing.

## Declaration of competing interest

The authors declare that they have no known competing financial interests or personal relationships that could have appeared to influence the work reported in this paper.

## Data Availability

No data was used for the research described in the article.

## Appendix Supplementary materials

Image, application 1
