# Supplementary material for: High throughput application of ASTM D8332: Detailed prototype design and operating conditions for microplastic sampling of riverine systems
Source: MethodsX. 2024 Mar 26;12:102680. doi: 10.1016/j.mex.2024.102680 (PMC10995887; doi:10.1016/j.mex.2024.102680)

**Supplementary Material**

1. Updated Wiring Schematic for Prototype Sampling System


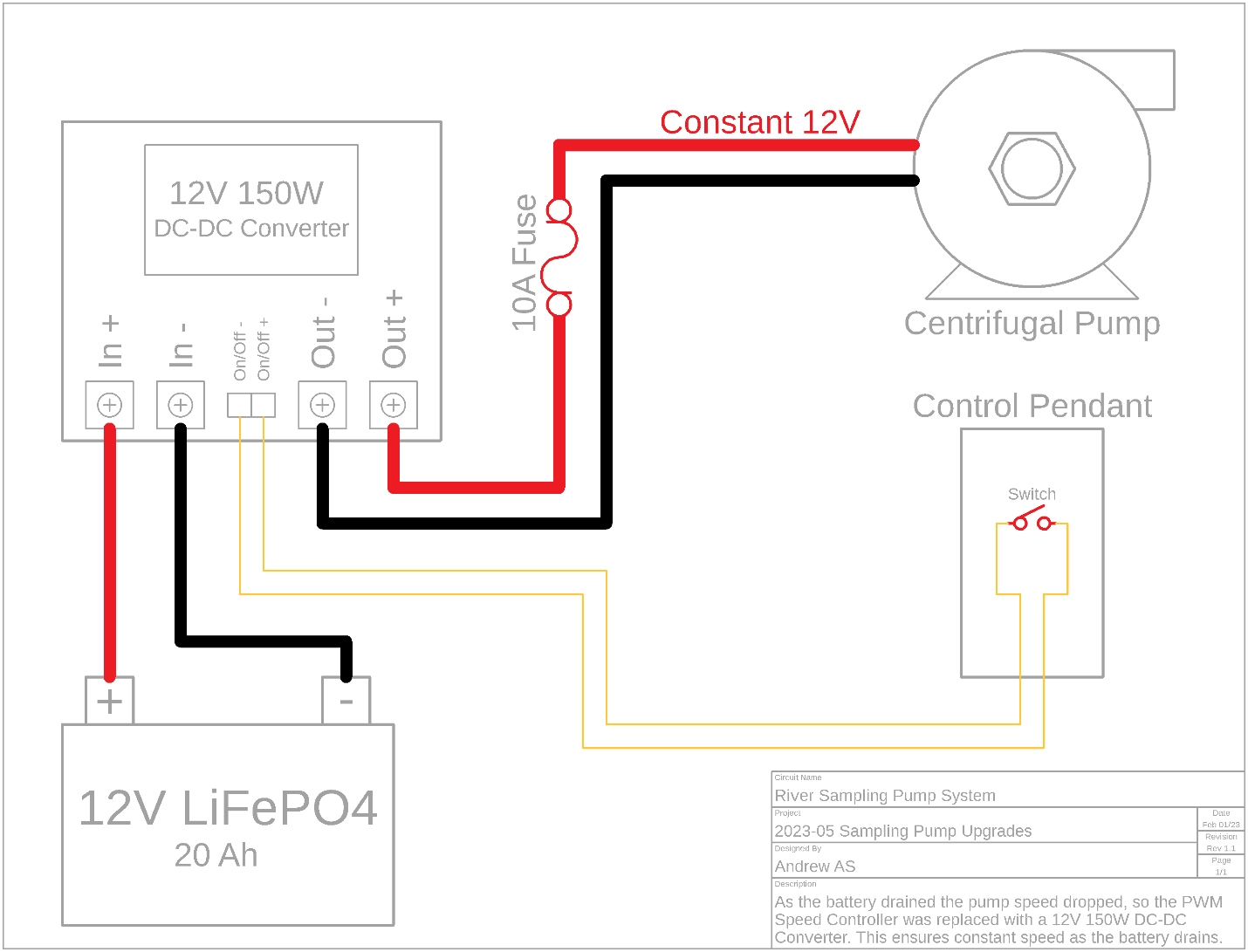


1. Bill of Materials (BOM) for Updated Wiring Schematic for Prototype Sampling System

| **Vendor** | **Part #** | **Description** | **Qty.** |
| --- | --- | --- | --- |
| Digikey | GRB112A802BB | On/Off Switch | 1 |
|  | CN5702 | Enclosure for Control Pendant | 1 |
|  | CQB150W-24S12 | 12V 150W DC-DC Converter Module | 1 |
|  | 1550WD | Enclosure for DC-DC Converter Module | 1 |
|  | AT04-3P-PM11 | Deutsch 3p Bulkhead Connector - Male Pins | 1 |
|  | AW3P | Deutsch 3p Pin Wedge | 1 |
|  | AT04-2P-PM11 | Deutsch 2p Bulkhead Connector - Male Pins | 1 |
|  | AW2P | Deutsch 2p Pin Wedge | 1 |
|  | AT60-202-16141 | Deutsch 16-20AWG Crimp Pin | 5 |
|  | AT06-3S-BLK | Deutsch 3p Cable Connector - Female Sockets | 1 |
|  | AW3S | Deutsch 3p Socket Wedge | 1 |
|  | AT06-2S-BLK | Deutsch 2p Cable Connector - Female Sockets | 1 |
|  | AW2S | Deutsch 2p Socket Wedge | 1 |
|  | AT62-201-16141 | Deutsch 16-20AWG Crimp Socket | 5 |
|  | 22003131-01 | Push-Twist Cable Connector - Male Pins | 2 |
|  | 22003231-01 | Push-Twist Cable Connector - Female Sockets | 2 |
|  | 22003535-03 | Push-Twist Bulkhead Connector - Male Pins | 2 |
|  | 22003635-03 | Push-Twist Bulkhead Connector - Female Sockets | 4 |
|  | 806-00053-00 | Bulkhead Cap | 6 |
| TME Electronics | 00100644 | 3 Conductor 16AWG Cable for Pump and Pendant | 10 |

1. Obsolete Wiring Schematic for Prototype Sampling System


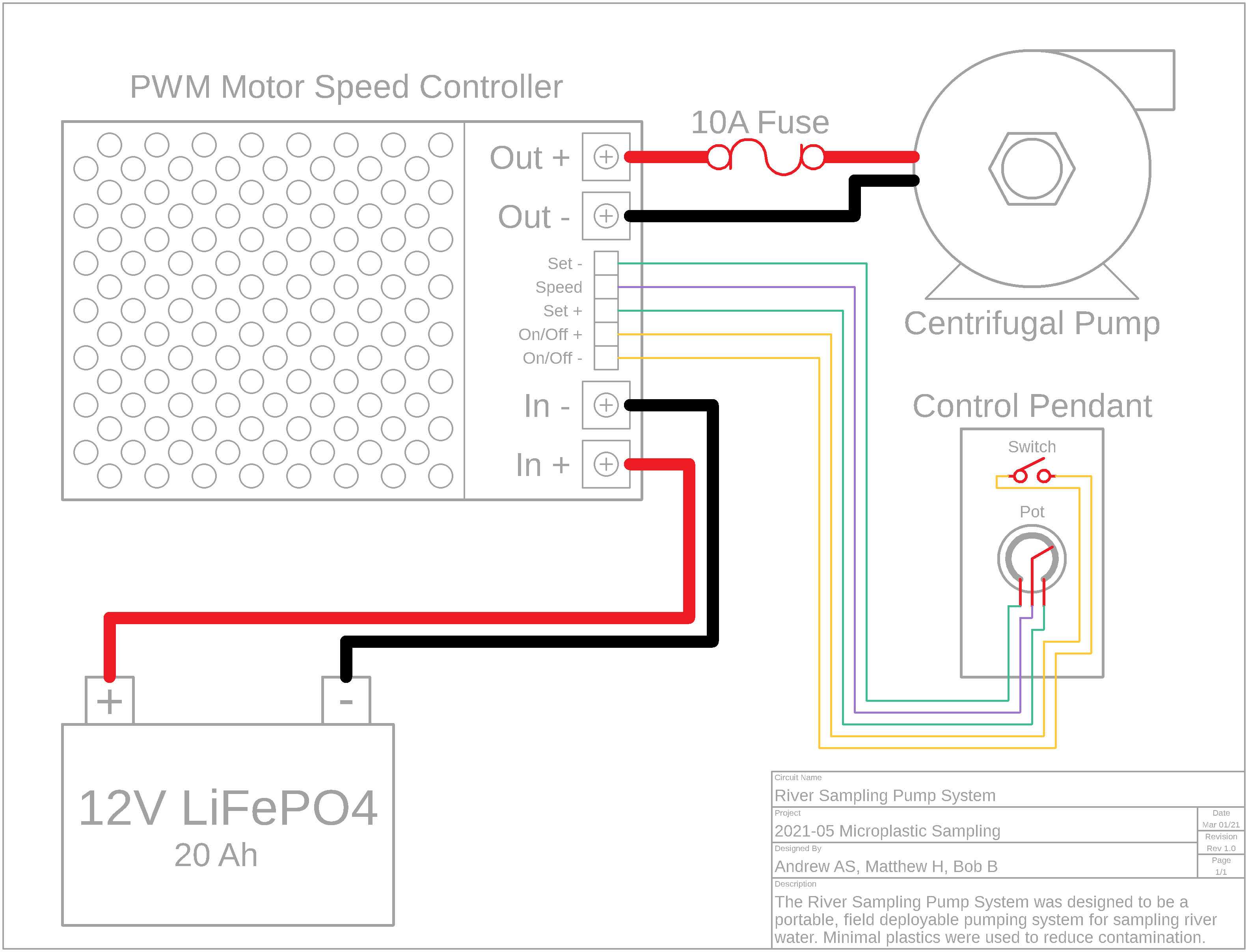

Supplement: Supplementary file 1 [file mmc1.docx]
